# Assessment and Modeling of the Influence of Age, Gender, and Family History of Hearing Problems on the Probability of Suffering Hearing Loss in the Working Population

**DOI:** 10.3390/ijerph17218041

**Published:** 2020-10-31

**Authors:** Jesús P. Barrero, Eva M. López-Perea, Sixto Herrera, Miguel A. Mariscal, Susana García-Herrero

**Affiliations:** 1 Escuela Politécnica Superior, University of Burgos, Avenida Cantabria s/n, 09006 Burgos, Spain; jpbarrero@ubu.es (J.P.B.); susanagh@ubu.es (S.G.-H.); 2 Facultad de Educación, University of Burgos, C/Villadiego 1, 09001 Burgos, Spain; emlperea@ubu.es; 3 Department of Applied Mathematics and Computer Sciences, University of Cantabria, 39005 Santander, Spain; herreras@unican.es

**Keywords:** hearing loss, Bayesian network, gender, age, family history

## Abstract

Hearing loss affects hundreds of millions of people all over the world, leading to several types of disabilities, ranging from purely physical to psychological and/or social aspects. A proper analysis to ascertain the main risk factors is essential in order to diagnose early and treat adequately. An exploratory analysis based on a heterogeneous sample of 1418 workers is presented in order to identify the main trigger factors for hearing loss. On the one hand, we recorded several medical and environmental parameters, and on the other, we created a model based on Bayesian networks in order to be able to infer the probability of hearing loss considering different scenarios. This paper focuses on three parameters: gender, age, and a family history of hearing problems. The results obtained allow us to infer or predict the best or worst auditory level for an individual under several different scenarios. The least relevant factor is the existence of a family history of deafness, followed by the gender factor, which slopes considerably toward better hearing for females, and most prominent of all, the age factor, given the large differences identified between the various age groups when the gender and family history of deafness variables remain constant.

## 1. Introduction

Hypoacusia is a prevalent condition in the population that affects some 360 million people worldwide, resulting in various levels of disability ranging from physical to social and psychological aspects [1]. In particular, hypoacusia could lead to disorders involving speech, resulting in difficulties taking part actively and effectively in conversations in different listening environments, to cognitive impairment, such as dementia, mental health problems, such as anxiety and depression [2,3,4,5], and even to an increased mortality risk [6,7]. There is consensus that depending on its degree or severity, the frequencies affected, and the stage of life in which it appears, hypoacusia can cause severe damage to the development of speech, language, and cognitive abilities, especially if it presents in prelingual children. Numerous studies have shown that 1–2 out of 1000 newborns have severe to profound hearing loss [1,8]. It has been shown that hearing impairment affects the progress of children in school, and later, their ability to obtain, hold, and perform a job. For all ages and both genders, the loss of hearing leads to problems with interpersonal communications and causes significant individual social problems, and especially isolation and stigmatization.

Fernando Pablo [9] identifies noise as the most usual trigger for this disease, especially in industrialized nations. In fact, it has been estimated that between 17% and 22% of the population of the European Union (approximately 80 million people) deals with sound levels above 65 dB [10]. The World Health Organization (WHO, Geneva, Switzerland) specified the tolerance limit of 65 dB. Either directly or indirectly, exceeding that tolerance limit is responsible for 11% of all workplace accidents. Thus, noise-related hypoacusia ranks third in terms of professional diseases [11,12] and, according to the World Health Organization (WHO, Geneva, Switzerland), it is also third among diseases involving years lived with disability (YLD) behind depression and unintentional injuries [1].

Although the most common trigger for this disease is noise [9], in terms of the risk factors for developing hypoacusia as a professional disease, the first-order factors can generally be considered to be physical characteristics such as sound pressure, the type of noise, and length of exposure as well as other factors such as age and some personal characteristics of the worker [9,13,14]. Other aspects to be considered are position relative to the sound source and the distance, the work environment, diseases, osteosclerosis, and gender, among others. It is necessary to study more variables from the safety management system [15]. In this study, we will focus on three of the factors presented above: age, gender, and a family history of deafness or significant hearing problems.

While one could assume that the ears of young people are more resistant due to their more elastic basilar membrane, there are no conclusive data to support this hypothesis. It is believed that the likelihood of an injury rises in middle-aged individuals, and a great deal of controversy surrounds the correlation that might exist with the hearing loss caused by age itself (presbycusis) [10]. Fernando Pablo [9] states that the ear deteriorates with age, and that depending on the type of noise exposure present, hearing loss will result. Then, there seems to be consensus that hearing worsens with age, which is a subject that various studies consider from different perspectives. One such researcher is Martín Villares [16], who has related presbycusis with hyperlipidemia and concludes that patients that presented with hyperlipidemia exhibited worse hearing based on the relationship between lipids and aging of the hearing canal caused by an atherogenic mechanism. It is also important to note that presbycusis is thought to be a biological phenomenon that affects everyone, starting at around 20 to 30 years of age and exhibiting its first symptoms between 50 and 60 years of age [2,6]. Thus, it may be regarded as a natural condition whose prevalence increases with age [1].

Although it is not predominant in either gender, various studies indicate that women are more resistant to noise than men [17] and thus have better hearing [18,19,20]. Similarly, Calviño del Río concludes that women are less sensitive to decreases in auditory thresholds [21]. Various existing studies relate age and gender jointly to hearing loss [22]. However, the studies are inconclusive in terms of the specific patterns in gender difference for age-related hearing loss [23]. One example is the study by Sharashenidze et al. [24], which states from the ages of 30–39 until 40–49 and 50–59, hearing loss is faster in men than women; in contrast, the same study indicates that starting at the ages of 60–69 and 70–79, hearing changes are more pronounced in women than in men. On the other hand, the study by Pearson et al. [23] states that hearing sensitivity decreases by more than twice in men as in women in most age and frequency ranges. Empirical research, such as that conducted by Ruiz [25], which studied AENA (Aeropuertos Españoles y Navegación Aérea) staff that worked at Los Rodeos Airport (Spain), concludes that a comparison of hearing thresholds between genders for each frequency shows that women lose hearing to a lesser degree than men, even when working in the same environment. However, this does not necessarily indicate that women are more resistant to noise-induced hearing loss than men, but rather that the women in the study were less exposed to loud noise levels than men. They also engage in fewer outside activities with loud noises, such as those experienced during hunting, which could explain the higher difference in hearing in the left ear, especially at those frequencies most affected by noise. In contrast, other authors note that at the same age, the hearing ability of women is lower and more susceptible than in men [26,27].

Finally, there are studies that consider the individual susceptibility to noise, which may be hereditary (with family history being relevant), and the risk factors associated with hypoacusia, such as ototoxic treatments, meningitis, diabetes mellitus, and high blood pressure, among others [1,10].

Bayesian networks have been extensively used in the field of healthcare [28,29,30,31,32,33] for disease diagnosis and transmission [34], system management [35], sensitivity analysis [36], and other applications. On the one hand, the Bayesian approach is employed in order to explicitly consider the uncertainty of the results [37,38] that are not easily interpretable or considered in other, more classical, methodologies (e.g., regression-based methods). On the other hand, Bayesian networks are able to represent and include complex, direct/conditional and linear/non-linear relationships between the variables considered [39,40], instead of direct bivariate dependences, as are considered in other approaches (e.g., regression-based methods). Finally, the directed acyclic graph (DAG) associated with the Bayesian network [41] allows the user to more easily interpret the dependences between the variables analyzed than by using more complex techniques, e.g., neural networks, multi-level/hierarchical path analysis [42,43].

This study analyzes the likelihood of developing hypoacusia, taking into account multiple variables. Bayesian networks (BN) are used. This model is based on demographic and personal factors, as well as occupational factors in different companies and non-work factors. The hypothesis is that the auditory health of people depends on the combination of all the aforementioned factors.

Lastly, it is important to note that the purpose of this study was to answer the basic questions of how and why hypoacusia develops in workers: What factors most influence hearing health in individuals? What effects do demographic factors or the physical characteristics inherent to the individual have, and to what extent? To answer these questions, our research specifically focuses on studying the key demographic variables (age and gender) in combination with the factor on family hearing of deafness and their influence on the development of hypoacusia.

## 2. Materials and Methods

### 2.1. Materials

Taking into account the data (medical and occupational) of a period of two years from the Servicio de Prevención Ajeno Ingemédica S.L. (Limited Society), we gathered a pool of employees in different sectors of activity. These individuals, of various ages and nationalities, work in companies of Spain.

The University of Burgos through its bioethics committee approved the study, and the ethical approval code is IR28/2020.

We first proceeded to take noise measurements in the workplaces of the individuals in our study. We also conducted audiometric tests, which were complemented by the surveys published and authorized by the Ministry of Health and Consumption [44]. All of these data were obtained with the consent of the workers, and companies and were kept anonymous and confidential.

As concerns the noise measurements, which were taken with sound level meters and dosimeters by qualified industrial hygiene specialists using proper measurement techniques, we were able to determine the sound levels in the different work environments in the companies where the workers in the sample carry out their activity. We also wrote personalized reports and evaluated the noise conditions at the companies, pursuant R.D. 286/2006, on protecting workers against the risks related to noise exposure [45].

In the sample, two steps were taken to assess the hearing ability of the workers. First, each individual was subjected to a pure-tone audiometry via air conduction, which yielded the target medical data on their hearing acuity. Second, the survey given to the participants included several questions on their habits as well as on the subjective perception that the respondents have of their hearing acuity.

As required by the applicable law on the Prevention of Occupational Risks [45,46], the audiometric tests were conducted using audiometers in sound-proof rooms by occupational physicians and medics.

The sample pool consisted of 1418 individuals, for whom we collected demographic data such as age, gender, nationality, etc.; occupational factors such as type of sector or activity, the noise level, the noise exposure, the use or non-use of hearing protection, occupational exposure to noise in previous jobs and exposure to ototoxic agents, time-exposure limits; and lastly, data on non-work factors, such as off-hours noise exposure (hunting, night clubs, etc.), a family history of hearing loss, use of drugs that affect hearing, and the history of auditory diseases or otologic events [14].

### 2.2. Conceptual Model

Our conceptual model aims to explain the development of hypoacusia due to different variables, personal and demographic, occupational and non-occupational.

To achieve this objective, we consider as key study variables the Speech Average Loss (SAL) index, the Early Loss Index (ELI), and the Overall Loss Percentage Index, which can most objectively determine the hearing acuity of an individual. We will analyze how these factors are affected by the other variables.

The variables involved in this model are:

- Personal and demographic variables: nationality, weight and height, blood pressure, gender, and age.
- Occupational variables: previous jobs (noise exposure in, years of occupational noise), noise level at workplace (measurement), daily hours of noise exposure, years at current job, occupational exposure to ototoxic agents, sector, workstation, and system for protecting against noise or use of personal protection.
- Non-occupational variables: off-hours exposure to noise, a family history of hearing problems, the presence of general diseases with the potential to affect hearing, a history of otologic events, and the use of ototoxic drugs.
- Hypoacusia evaluation variables: objective such as SAL index/ELI index/% Overall loss; or subjective such as hearing perception, quality of communications, Television volume, hearing ability in noisy environment, and sensitivity to loud noise.

### 2.3. Study Variables

The variables chosen for this study are as follows: the Percentage of Binaural Loss Index as an objective variable, the effects on which were considered for the age, gender, and a family history of hearing problems for the individuals in the sample.

#### 2.3.1. Percentage of Binaural Loss

The percentage of binaural loss (%Bl) is used for evaluating hearing acuity based on audiometric tests [13,44] that rely on a “social” evaluation of hearing loss. It is an index of considerable legal importance for assessing hearing impairment or hypoacusia that is used in Spain to obtain, pursuant to R.D. 1971/999 [47], the various disability percentages related to the level of hearing loss.

Hearing loss is evaluated by measuring the preliminary thresholds for 500 Hz, 1000 Hz, 2000 Hz, and 3000 Hz tones (see Equation (1)). In one ear, the loss percentage is the individual percentages associated with each tone. To know the general loss percentage in both ears, the loss in the better ear (expressed as a percentage of hearing loss) is multiplied by 5, and it is multiplied by 1 for the worse ear. Then, the losses are added and divided by 6, as shown in Equation (2) (Equations for calculating overall loss percentage. Source: Uña Gorospe M.A.)

(1) $$monauralloss=\left(\frac{\sum LossdB\left(A\right)\;for\;500,\;1000,\;2000,\;3000\;\mathrm{Hz}}{4}-25\right)\times 1.5$$

(2) $$binauralloss=\left(\frac{5\times loss\in thebetterear+loss\in theworseear}{6}\right)$$

**Figure 1:**
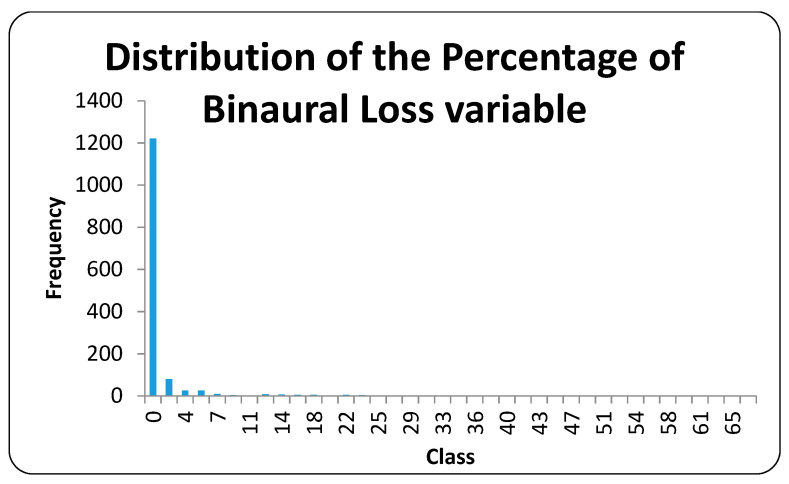
Distribution of the percentage of binaural loss variable. Source: Compiled by authors.

Figure 1 shows the sample distribution based on the percentage of binaural loss of the individuals. The maximum value is 67%, and the minimum value is 0%. The average binaural loss is 1%.

The variable was discretized on the following intervals (see Table 1).

#### 2.3.2. Gender

The gender variable refers to the sex and is therefore an independent, qualitative, and nominal variable. The sample pool consists of 1233 men and 185 women, which represents 86.95% and 13.05% of the sample, respectively.

#### 2.3.3. Age

**Figure 2:**
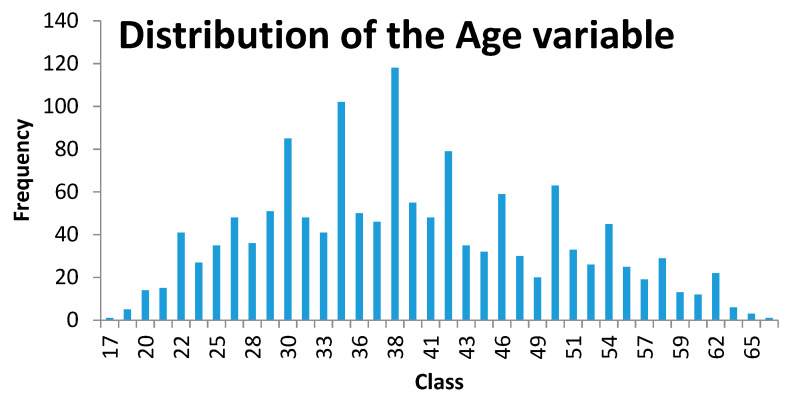
Distribution of the age variable. Source: Compiled by authors.

The distribution of the sample pool based on the age of the individuals is shown in Figure 2. The average age is 38, with a minimum of 17 and a maximum of 66.

This variable was discretized into quintiles for statistical processing, yielding the following groups based on age ranges (see Table 2).

#### 2.3.4. Family History of Hearing Problems

This variable is divided into just two groups, depending on whether or not the worker had a family history of deafness or other significant otorhinolaryngological conditions (see Table 3).

### 2.4. Bayesian Networks (BN)

**Figure 3:**
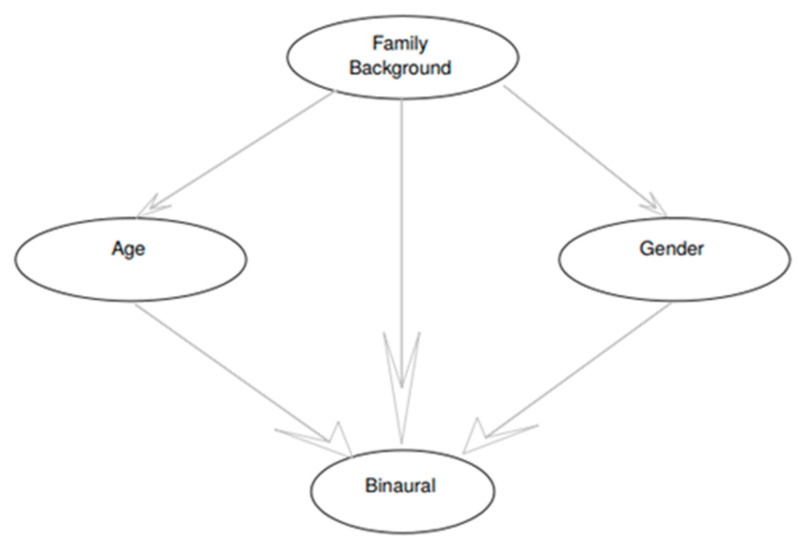
Subgraph of the Bayesian network. Source: Compiled by authors.

**Figure 4:**
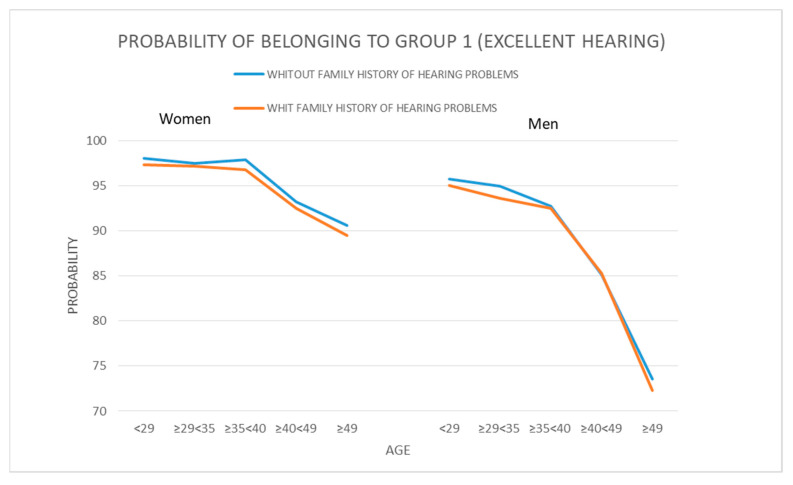
Graph comparing the probability of excellent hearing by gender, age, and family history of hearing problems. Source: Compiled by authors.

Bayesian networks (BNs) [48] are probabilistic graphical models [41] based on a directed acyclic graph (DAG), see e.g., Figure 3. The DAG describes dependences, conditioned or not, between the variables considered in the model. These dependences are used to simplify the factorization of the joint probability distribution (JPD) in terms of conditioned probabilities: $p\left(x_{1,}x_{2,}\ldots ,x_{n}\right)=\prod_{i=1}^{n}p\left(x_{i}|\pi_{i}\right)$, where $\pi_{i}$ corresponds to the parents of $x_{i}$ in the DAG. As an example, in the graph shown in Figure 4, the number of parameters needed to define the JPD, in the case of binary variables (1 or 0), is 2^4^ − 1 = 15. However, considering the DAG, only 4 parameters are needed, which will simplify the learning process.

As we have seen, in order to train a Bayesian network, two steps are needed. First, the DAG has to be trained (known as structural learning), and secondly, the conditioned probabilities of the factorization obtained have to be adjusted to yield the final JPD. Both processes can be done automatically considering the data available [49]. This results in a JPD that can, by applying efficient algorithms, be used, together with the graph, to infer the new probabilities once any new evidence or knowledge appears.

### 2.5. Model Performance. Receiver-Operating Characteristics

Note that, for each group of the target variable, a natural classifier is obtained defining a threshold for the probability given by the Bayesian network above (below) which the corresponding group occurs (or not). As a result, for each group, this classifier should be properly evaluated to both know the accuracy of the obtained model and avoid possible overfitting to the training sample, which leads to lower than expectable accuracy for new samples. In order to do this, a 10-fold cross-validation [50] is proposed by dividing the full sample into 10 disjoint data subsets, containing *N*/10 elements each. The data subsets will be used once as a test set, and the remaining data will be used to train the model [13]. Note that by joining the 10 folds, a prediction for the whole is obtained that could be compared to the data. For each fold and the joined prediction, the Area Under the ROC (Receiver Operating Characteristic [51]) Curve (AUC) was obtained, which is a standard validation approach for probabilistic and binary classifiers. This parameter varies from 0.5 (random guess) to 1 (perfect performance) and can be interpreted as a measure of overall accuracy [52]. An AUC has been obtained for the target variable of each group. The results obtained in this study were 0.96, 0.95, 0.98, 0.87, and 0.75, showing a high overall accuracy of the 5 groups.

## 3. Results

In this section, we summarize the main results of the analysis. First, we will describe the BN and the marginal probabilities. The latter have been adjusted for the primitive variables (ages, gender, and family history) to analyze their influence on the hearing health of the sample considered. Then, the results of the sensitivity analysis of the primitive variables are described in the subsections that follow.

### 3.1. Bayesian Network Utilized

Below is the subgraph resulting from the proposed Bayesian network, showing the relationship between the primitive variables (see Figure 3).

Table 4 shows the initial probabilities of the primitive variables. The results show that most individuals have good hearing, with no or minor hearing loss (Groups 1 and 2, respectively). Considering the different variables analyzed, we see that the women exhibit higher hearing acuity than men, with 96.08% of them exhibiting the best hearing possible, versus 87.95% of men. As for the age, the probability of having good hearing is highest for young people, at 95.97%, gradually descending with increasing age to 74.83% for the group of people older than 49. Finally, Table 4 shows that those individuals with no family history of hearing problems are 89.26% likely to belong in Group 1 (the best possible hearing) versus 87.25% of individuals with hearing loss.

### 3.2. Sensitivity Analysis. Gender and Age vs. Percentage of Binaural Loss Index

This study considers the joint influence of gender and age as factors that affect the development of hypoacusia (see Table 5). It must be noted that women’s hearing acuity is always better than men’s, when their respective age groups are compared. Table 5 also shows that the likelihood of belonging to the group with the best hearing (Group 1) is also higher in women (above the initial 89.04%), regardless of their age. Even in the worst case scenario, i.e., women ≥49 years of age, are still 90.47% likely to belong to the best hearing group. Table 5 also shows that men over the age of 49 have the lowest probability of being in the group with the best hearing, Group 1 (73.42%).

Thus, it may be concluded that hearing loss increases with age, which is an effect that is more pronounced in men.

### 3.3. Sensitivity Analysis. Gender and Family History of Hearing Problems vs. Percentage of Binaural Loss Index

The aim of this study is to determine the combined influence of gender and family history of hearing problems variables in the development of hypoacusia. We see that in both women and men, the probabilities of belonging to the group with the best hearing (Group 1, no loss of hearing acuity) is higher for individuals who do not report a family history of hearing problems than for those who do (see Table 6). Once more, women in general have a higher probability of exhibiting good hearing than men, whether or not they report a family history of hearing problems. In fact, the probability of belonging to the group with the best possible hearing is 94.86% in the least favorable case for women (i.e., women with family history of hearing problems), versus 88.14% in the most favorable case for men,(i.e., men with no family history of hearing problems).

### 3.4. Sensitivity Analysis. Age and Family History of Hearing Problems vs. Percentage of Binaural Loss Index

This study shows that people with no family history of hearing problems are more likely to belong in the group with the best hearing, for every age group (Table 7). We can also see in this case the decisive influence of age, such that the probability of having the best hearing possible falls gradually with increased age, from 96.04% or 95.19% for the age range containing the younger individuals, to 75.04% or 73.31% for the age range containing those older than 49.

### 3.5. Sensitivity Analysis. Family History of Hearing Problems, Gender and Age vs. Percentage of Binaural Loss Index

Table 8 shows the joint influence of the three variables (family history of hearing problems, gender, and age) on the development of hypoacusia.

As far as the family history of hearing problems factor is concerned, we see that for people of the same gender and age, the likelihood of being in the Group 1 (best hearing) is higher for individuals who do not report a family history of hearing problems than for those who do. The difference in these probabilities is around one percentage point. For example, in the case of women ranging in age between 35 and 40, the likelihood of being in Group 1 is 97.92% when no family history of hearing problems is reported, versus 96.78% when such a history is present.

As for the gender factor, we see that hearing in the women’s group is always better than in the men’s group when the age and family history of hearing problems factors are the same. The differences in the probability of belonging in the group with the best hearing are around 3 percentage points for the youngest individuals in the sample, and they rise to 17 percentage points for the older individuals. For example, we see that for women 49 years of age and older that do not report a family history of hearing problems, the probability is 90.56%, while for men of the same age who also report no family history of hearing problems, this probability is 73.58%.

Focusing now on age, we see than in every case, regardless of whether or not a family history of hearing problems is reported, hearing acuity drops as the age of the individuals rises. In the case of women, the differences between the probabilities of being in Group 1 is 8 percentage points between the youngest and oldest individuals, which is a difference that rises to 23 percentage points in the case of men. For example, for men who report a family history of hearing problems, the probability is 95.03% for those under the age of 29 and 72.29% for those over the age of 49.

In view of the results, we can conclude that the variables analyzed affect the development of hypoacusia such that the likelihood of presenting this condition increases with age, especially for men and if a family history of significant hearing problems is reported (see Figure 4).

Consequently, the influence of the three variables analyzed varies in intensity, such that the element with the least effect, meaning the factor that exhibits the lowest percent variation when the other two factors are unchanged, is the existence of a family history of deafness, followed by gender, which slopes considerably toward better hearing for females, and most prominent of all, age, which is the most crucial of all given the large differences identified between the various age groups when the variables involving gender and family history of deafness or significant hearing problems remain constant.

## 4. Discussion

The first result of our research was the compilation of an extensive database, useful for multiple studies, that allows us to infer or predict the best or worst hearing acuity for an individual given several different scenarios. By creating a Bayesian network based on the data obtained from a sample of 1418 workers and consistent with the main factors responsible for hypoacusia, classified by demographic/personal origin and occupational and non-occupational variables, we were able to more accurately weigh and ascertain those variables that are most influential in the development of hypoacusia. One of the main findings of this study lies in the use and operation with the aforementioned Bayesian network methodology to conduct a combined and predictive analysis of how the factors of gender, age, and family history of hearing problems affect the probability that an individual will exhibit good or poor hearing acuity.

The hearing acuity was assessed via medical audiometric tests that were processed using the criterion of the Percentage of Binaural Loss Index, which is an intuitive and widely used method, of critical importance to the legal process of quantifying disability due to deafness of hypoacusia. For our sample pool, we determined that the average binaural loss was 1%. Taking into consideration the magnitude of the sample, which consisted of 1418 workers in various economic sectors and different jobs, we found their auditory health to be generally good, as there were only 6 people (0.28%) who showed hearing loss of more than 30% versus 86.11% of people who did not show any hearing loss at all.

Our study of the factors that influence an individual’s hearing acuity first analyzed the three selected factors—gender, age, and family history of hearing problems—separately. We noted that women exhibit better hearing acuity than men. As for age, the younger respondents (below the age of 29) are more likely to have good hearing, which is a probability that decreases inversely with age. Lastly, as for the family history of hearing problems, those individuals who do not report such a history have a slightly higher likelihood of having better hearing than those who do indicate some type of family history.

Then, we analyzed the combined influence of the gender and age factors, noting that hearing in women is always better than in men when their respective age groups are compared. We also concluded that women, regardless of their age, are more likely to be in Group 1 (best hearing) and that this probability always exceeds the initial probability of 89.04%.

As concerns the analysis of the influence that the gender and family history of hearing problems variables have on the development of hypoacusia, we see that in both women and men, the probability of having good hearing is higher for those who do not report a family history of hearing problems than for those who do.

When we both studied the influence of the age and family history factors, we saw that for every age range, those with the highest likelihood of having good hearing are those who do not report a family history of hearing problems.

A study of all three factors—gender, age, and family history—combined confirms the above findings. As for the factor involving a family history of hearing problems, we see that for individuals of the same age and gender, the probabilities of being in the group with the best hearing are higher for those who do not report a family history of hearing problems than for those who do. As concerns the gender factor, we see that hearing acuity for women is always better than for men given the same age and family history of hearing problems. If we focus on age, we see that in every case, regardless of whether or not a family history of hearing problems is reported, hearing acuity decreases as the age of the individual increases.

In view of these results, we may conclude that the variables analyzed affect the development of hypoacusia, which is more likely to develop with age, especially in males, and more so if the individual reports a family history of deafness or considerable hearing problems.

As for the gender of the individuals, our study confirms the theories that ascribe a superior hearing acuity to women compared with men, such as those by Muñiz [17], Flodgren [18], and Hallmo [20], and that they are less prone to declining hearing thresholds, as reported by Calviño del Río [21]. Our results may also be interpreted to indicate that women lose their hearing to a lesser extent than men. However, they do not necessarily indicate that women are more resistant to noise-induced hearing loss than men; rather, they indicate that women are generally less exposed to loud noise levels than men, as was concluded by Ruiz [25]. In future research work, it would be good to carry out this same study including as a variable the workplace.

In terms of the age factor, our work substantiates those studies that find that hearing worsens with age, that the ear deteriorates with age, and that independent of the type of noise exposure experienced, hearing loss will result, as concluded by Fernando Pablo [9]. It also seems to confirm that presbycusis is a biological phenomenon to which no one is immune (Lin [2] and Yueh [6]) and could be regarded as a natural condition that becomes more prevalent with age, as noted by Díaz [1].

As concerns the family history of hearing problems and its effect on developing hypoacusia, our study supports the findings of Sanz [10], who underscores the individual susceptibility to noise, which might be hereditary, among other factors that trigger hypoacusia. Díaz [1] expressly includes a family history of hearing problems as a risk factor associated with hypoacusia.

It is important to emphasize that the three variables analyzed have different levels of influence on a person’s hearing, such that the least relevant variable is the existence of a family history of deafness, followed by the gender variable, which varies considerably in favor of better hearing for females, and most prominent of all, the age variable, given the large differences identified between the various groups, while the gender and family history of deafness variables remain constant.

## Figures and Tables

**Table 1:** Binaural loss sample distribution. Source: Compiled by authors.

Group	Binaural Loss	No. of Cases	Frequency
I	0%	1221	86.11%
II	15% ≥ X > 0%	163	11.50%
III	30% ≥ X > 15%	28	1.97%
IV	45% ≥ X > 30%	4	0.28%
V	X > 45%	2	0.00%
	TOTAL	1418	100%

**Table 2:** Sample distribution by age. Source: Compiled by authors.

Group	Age Range	No. of Cases	Frequency %
1	<29	273	19
2	≥29 < 35	276	20
3	≥35 < 40	269	19
4	≥40 < 49	303	21
5	≥49	297	21
	Total	1418	100

**Table 3:** Sample distribution based on family history of hearing problems. Source: Compiled by authors.

Group	Family History of Hearing Problems	No. of Cases	Frequency %
1	No	1239	87.38
2	Yes	179	12.62
	Total	1418	100

**Table 4:** Initial probabilities of the variables vs. Percentage of Binaural Loss Index. Source: Compiled by authors.

Initial Probabilities						
Variables		Groups of Binaural Loss Index				
		Group 1	Group 2	Group 3	Group 4	Group 5
		(0%)	(≥0 < 15%)	(≥15 < 30%)	(≥30 < 45%)	(≥45%)
Hypoacusia	Initial percentage	89.04	9.89	1.01	0.06	0.00
Gender	Women	96.08	3.74	0.18	0.00	0.00
	Men	87.95	10.84	1.14	0.07	0.00
Age	<29	95.97	3.86	0.00	0.17	0.00
	≥29 < 35	95.25	4.52	0.23	0.00	0.00
	≥35 < 40	93.75	6.22	0.03	0.00	0.00
	≥40 < 49	85.93	12.93	1.12	0.02	0.00
	≥49	74.83	21.38	3.67	0.12	0.00
Family history of hearing problems	No	89.26	9.65	1.03	0.06	0.00
	Yes	87.25	11.87	0.84	0.04	0.00

**Table 5:** Sensitivity analysis. Gender and age vs. Binaural Percentage Index. Source: Compiled by authors.

Variables		Groups of Binaural Loss Index				
		Group 1 (0%)	Group 2 (≥0 < 15%)	Group 3 (≥15 < 30%)	Group 4 (≥30 < 45%)	Group 5 (≥45%)
Initial percentage of hypoacusia		89.04	9.89	1.01	0.06	0.00
Gender	Age					
Women	<29	98.01	1.99	0.00	0.00	0.00
	≥29 < 35	97.46	2.15	0.38	0.00	0.01
	≥35 < 40	97.84	2.16	0.00	0.00	0.00
	≥40 < 49	93.15	6.56	0.29	0.00	0.00
	≥49	90.47	9.27	0.25	0.01	0.00
Men	<29	95.69	4.11	0.00	0.19	0.00
	≥29 < 35	94.80	5.01	0.20	0.00	0.00
	≥35 < 40	92.68	7.29	0.04	0.00	0.00
	≥40 < 49	85.13	13.64	1.21	0.02	0.00
	≥49	73.42	22.47	3.98	0.13	0.00

**Table 6:** Sensitivity analysis. Gender and family history of hearing problems vs. Binaural Percentage Index. Source: Compiled by authors.

Variables		Groups of Binaural Loss Index				
		Group 1	Group 2	Group 3	Group 4	Group 5
		(0%)	(≥0 < 15%)	(≥15 < 30%)	(≥30 < 45%)	(≥45%)
Initial percentage of hypoacusia		89.04	9.89	1.01	0.06	0.00
Gender	Family history of hearing problems					
Women	No	96.17	3.65	0.18	0.00	0.00
	Yes	94.86	4.98	0.12	0.00	0.03
Men	No	88.14	10.62	1.17	0.07	0.00
	Yes	86.52	12.53	0.91	0.04	0.00

**Table 7:** Sensitivity analysis. Age and family history of hearing problems vs. Binaural Percentage Index. Source: Compiled by authors.

Variables		Groups of Binaural Loss Index				
		Group 1 (0%)	Group 2 (≥0 < 15%)	Group 3 (≥15 < 30%)	Group 4 (≥30 < 45%)	Group 5 (≥45%)
Initial percentage of hypoacusia		89.04	9.89	1.01	0.06	0.00
Age	Family history of hearing problems					
<29	No	96.04	3.78	0.00	0.19	0.00
	Yes	95.19	4.81	0.00	0.00	0.00
≥29 < 35	No	95.38	4.38	0.24	0.00	0.00
	Yes	93.99	5.85	0.14	0.00	0.02
≥35 < 40	No	93.82	6.15	0.03	0.00	0.00
	Yes	93.15	6.81	0.04	0.00	0.00
≥40 < 49	No	85.95	12.92	1.11	0.02	0.00
	Yes	85.82	13.03	1.13	0.02	0.00
≥49	No	75.04	20.98	3.87	0.12	0.00
	Yes	73.31	24.27	2.28	0.14	0.00

**Table 8:** Sensitivity analysis. Family history of hearing problems, gender, and age vs. Binaural Percentage Index. Source: Compiled by authors.

Variables			Groups of Binaural Loss Index				
			Group 1 (0%)	Group 2 (≥0 < 15%)	Group 3 (≥15 < 30%)	Group 4 (≥30 < 45%)	Group 5 (≥45%)
Initial percentage of hypoacusia			89.04	9.89	1.01	0.06	0.00
Family history of hearing problems	Gender	Age					
No	Women	<29	98.04	1.96	0.00	0.00	0.00
		≥29 < 35	97.48	2.13	0.39	0.00	0.00
		≥35 < 40	97.92	2.08	0.00	0.00	0.00
		≥40 < 49	93.22	6.47	0.31	0.00	0.00
		≥49	90.56	9.18	0.25	0.01	0.00
	Men	<29	95.76	4.03	0.00	0.21	0.00
		≥29 < 35	94.93	4.86	0.21	0.00	0.00
		≥35 < 40	92.70	7.27	0.04	0.00	0.00
		≥40 < 49	85.10	13.67	1.21	0.02	0.00
		≥49	73.58	22.09	4.21	0.13	0.00
Yes	Women	<29	97.35	2.65	0.00	0.00	0.00
		≥29 < 35	97.14	2.39	0.31	0.00	0.16
		≥35 < 40	96.78	3.22	0.00	0.00	0.00
		≥40 < 49	92.48	7.43	0.09	0.00	0.00
		≥49	89.49	10.23	0.25	0.03	0.00
	Men	<29	95.03	4.97	0.00	0.00	0.00
		≥29 < 35	93.62	6.26	0.12	0.00	0.00
		≥35 < 40	92.52	7.42	0.05	0.00	0.00
		≥40 < 49	85.31	13.46	1.21	0.02	0.00
		≥49	72.29	25.15	2.41	0.14	0.00
